# Contributions of vascular ageing to late‐onset Alzheimer's disease

**DOI:** 10.1113/EP092909

**Published:** 2026-04-09

**Authors:** Skylyn J. Ferguson, Young Deanna Choi, Ashley E. Walker

**Affiliations:** ^1^ Department of Human Physiology University of Oregon Eugene Oregon USA

**Keywords:** amyloid, cerebral blood flow, cerebrovascular, dementia, endothelial

## Abstract

Late‐onset Alzheimer's disease (LOAD) is an age‐related disease that is strongly associated with vascular risk factors and cerebrovascular impairments. As such, changes in the vasculature with advancing age likely contribute to LOAD, but the mechanisms underlying these contributions remain incompletely understood. With advancing age, there is dysregulation of cerebral blood flow, impairment of neurovascular coupling, and increased blood–brain barrier permeability, which may initiate or contribute to the neuropathology associated with LOAD. Changes to the vasculature outside of the brain, including increases in blood pressure and arterial stiffness, may initiate age‐related cerebrovascular impairments. Age‐related increases in oxidative stress and inflammatory signalling, as well as contributions to LOAD‐related neuropathology, such as amyloid‐β and hyperphosphorylated tau, impair cerebrovascular cells. In this review, we summarize the evidence for the role of vascular ageing in LOAD, describing age‐related cerebrovascular impairments and their causes.

## Abstract (highlights)

- **What is the topic of this review?**
  While advancing age and vascular impairments are known to be associated with late‐onset Alzheimer's disease (LOAD), the specific contributions of vascular ageing to the disease are incompletely understood. This review offers a unique perspective on how age‐related changes to the vasculature, in the brain and beyond, may impact LOAD.
- **What advances does it highlight?**
  The review discusses traditional LOAD pathologies, such as amyloid‐β and tau, as well as mechanisms specific to the vasculature. Furthermore, this review considers evidence from both clinical and preclinical studies, providing mechanistic and translational insights to further our understanding of LOAD.

## 1 Introduction

The brain requires substantial support from the cardiovascular system, and age‐related changes to the vasculature have devastating consequences for brain function. The brain needs a large amount of blood flow relative to its size, positioning it as an organ most affected by changes to vascular function (Iadecola & Gottesman, 2018). At the same time, the cerebral circulation requires a tight regulation of blood flow and pressure. Failure to meet the vascular demands of the brain can trigger neuroinflammation and the accumulation of aggregated proteins (Iadecola & Gottesman, 2018). As such, declines in vascular function with age can contribute to the onset or progression of neurodegenerative disease, such as Alzheimer's disease (AD).

Dementia is an umbrella term that refers to multiple diseases characterized by cognitive decline that arise from distinct pathologies and brain regions. Often, pathology assessment indicates a mixture of dementias present in the brains of older adults, such as a combination of features of AD pathology and vascular pathology (Kapasi et al., 2017). AD is classified as having an accumulation of biomarkers such as amyloid‐β (Aβ) proteinopathy or hyperphosphorylated tau, associated with neurodegeneration and neural injury. Late‐onset AD (LOAD) is the form of the disease that occurs after the age of 65 years and comprises 95% of cases (Masters et al., 2015). Distinct from AD is vascular dementia, more recently referred to as vascular cognitive impairment (VCI) to include a wider range of impairments, which results from cerebrovascular brain injury, such as post‐stroke infarct and subcortical ischaemia (Iadecola et al., 2019). However, VCI is not the only dementia influenced by vascular impairment. Recognizing that vascular factors contribute to many dementias, these are classified under the umbrella term ‘vascular contributions to cognitive impairment and dementia’ (VCID) (Zlokovic et al., 2020). Age‐related changes to the vascular system are a primary source of VCID, particularly as it relates to LOAD.

In this review, we will discuss the primary cerebrovascular functions that decline with advancing age. We will further discuss the intra‐ and extracranial contributors to these cerebrovascular declines, with a specific focus on those related to LOAD. VCI has been broadly reviewed elsewhere (Gorelick et al., 2011; Iadecola et al., 2019); therefore, we will not focus on impairment related to stroke/infarct in this review. Rather, in this review, we will focus on how changes to the ageing vasculature are specifically related to LOAD.

## 2 Cerebrovascular deficits with ageing and LOAD

Age‐related declines in cerebrovascular function result in the poor delivery of oxygen and nutrients and reduced removal of waste from the brain. These deficits manifest as lower or maldistributed blood flow, a loss of blood–brain barrier (BBB) integrity, and impairments to venous drainage or the glymphatic system. Dysregulation of cerebral blood flow (CBF) is found with advancing age and LOAD. With advancing age, there are declines in CBF, although this decline might be dependent on the presence of Aβ (Lu et al., 2011; Bangen et al., 2017). Furthermore, dysregulation of CBF is among the earliest predictors of LOAD, with CBF changes observed before metabolic changes, neuropathology, or cognitive impairment (Ruitenberg et al., 2005; Iturria‐Medina et al., 2016). For LOAD, the findings of CBF dysregulation are highly heterogeneous within disease stages and patient groups. At the very early stages of LOAD, before the onset of cognitive dysfunction and brain atrophy, CBF increases in patients (Fazlollahi et al., 2020). This counterintuitive increase in CBF in early LOAD is hypothesized to be a compensatory response to the presence of Aβ (Fazlollahi et al., 2020). Other studies have found that CBF is lower in AD patients, even in the early stages of the disease (Prohovnik et al., 1988). Lower CBF at baseline was associated with faster cognitive decline and increased development of dementia in patients with VCI (van Dinther et al., 2024).

While global CBF is an important metric of cerebrovascular health, more meaningful to brain function is the delivery of blood to working regions. The ability for CBF to increase in response to vasoactive stimuli is known as cerebrovascular reactivity (CVR), often measured as the response to hypercapnia. CVR declines with advancing age and LOAD (Alwatban et al., 2019; Sur et al., 2020; Yew et al., 2022). Impaired CVR is related to cognitive decline in healthy older adults and AD patients (Cantin et al., 2011; Peng et al., 2018). Physiologically, vasodilation occurs near active neurons to secure the delivery of oxygen and nutrients, a process known as neurovascular coupling (NVC) (Stackhouse & Mishra, 2021). Neurotransmitter release from neurons results in vasoactive signalling, which is largely mediated by astrocytes (Stackhouse & Mishra, 2021). NVC is impaired with advancing age and is related to cognitive decline (Mukli et al., 2023). The consequence of the mismatch between supply and demand for nutrients and oxygen is impaired neural activity, and NVC impairment precedes cognitive decline in AD mouse models (Lourenço et al., 2017). In short, CVR and NVC are pivotal elements of CBF regulation, and damage to these has been shown to be detrimental in both human and animal studies.

Cerebrovascular impairments with ageing and LOAD also impact what enters and exits the brain. Endothelial cells form the BBB, the tightest vascular barrier in the body. BBB impairment during healthy ageing occurs first in the hippocampus, a region vital for learning and memory (Montagne et al., 2015). A more permeable BBB allows proteins such as albumin and fibrinogen to enter the brain and accumulate, triggering neuroinflammation (Yang et al., 2020). As important as delivering nutrients to the brain is the removal of waste, particularly Aβ. The accumulation of Aβ could be due to insufficient venous drainage. A narrowing of the venous system is associated with cognitive impairment (Pardo et al., 2025). Waste is also removed from the brain along the vascular walls via the glymphatic system (Iliff et al., 2012). It has been proposed that the distension of cerebral arterioles creates the mechanical force to move fluid through the periarterial spaces. While the glymphatic system has been shown to clear Aβ in older adults without cognitive impairment (Dagum et al., 2026), this has not been studied in humans with LOAD. Thus, BBB integrity and the brain's waste removal systems are important for regulating the entry and removal of substances from the brain, but further evidence is needed for their roles in LOAD.

## 3 Cellular impairments with cerebrovascular ageing

Endothelial cells are responsible for controlling blood flow and facilitating fluid exchange. When endothelial cells are subjected to shear stress via blood flow, they produce vasodilatory signals. The most prominent vasodilatory signalling pathway is the upregulation of endothelial nitric oxide synthase (eNOS), resulting in the production of nitric oxide (NO) (Rubanyi et al., 1986). As NO is the primary substance responsible for endothelium‐dependent vasodilation, its bioavailability serves as a crucial marker of cerebrovascular health. Impaired cerebral artery endothelium‐dependent vasodilation is present in old age (Walker et al., 2014) and in AD rodent models (Tian et al., 2023). Furthermore, endothelial dysfunction contributes to impairments in NVC, including age‐related declines in NO bioavailability (Tarantini et al., 2017). In addition to endothelial cells, other cells of the neurovascular unit contribute to the regulation of blood flow.

**FIGURE 1:**
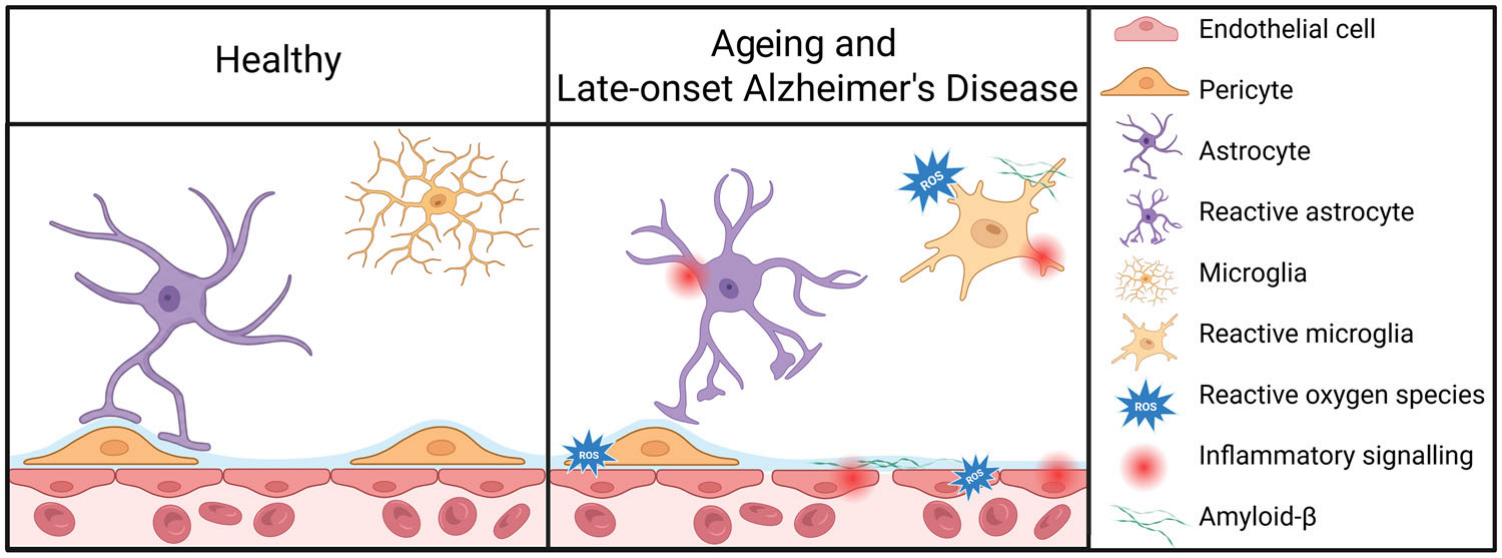
Mechanisms for cerebral microvascular impairment with ageing and late‐onset Alzheimer's disease. Left: a healthy cerebral microvasculature includes a tight endothelial cell barrier that is supported by pericytes and astrocyte endfeet. Right: with ageing and late‐onset Alzheimer's disease, there is an increase in amyloid‐β (Aβ), reactive oxygen species, and inflammatory signalling. These processes trigger endothelial cell dysfunction, leading to increased barrier permeability. A heightened inflammatory state leads to the activation of astrocytes and microglia. Additionally, there is a decrease in astrocyte endfeet interacting with the blood vessel and a loss of pericytes. Image created with BioRender.

Endothelial cells work together with pericytes, glial cells and neurons to form the neurovascular unit (Stackhouse & Mishra, 2021). Pericyte loss occurs with ageing and in cognitive decline, associated with reduced brain capillary density and impaired NVC (Bell et al., 2010). With ageing, astrocytes also develop a more reactive phenotype (Clarke et al., 2018), which may further induce neuroinflammation, while also leading to less support for NVC. The cells of the neurovascular unit are important not only for the regulation of blood flow but also for maintaining vascular barriers. The impairment of the BBB is partly due to a breakdown of endothelial tight junction proteins, such as claudin‐5, as well as pericyte migration (Montagne et al., 2015; Ting et al., 2023). Endothelial cell senescence also occurs with ageing (Rossman et al., 2017) and is associated with BBB leakage (Ting et al., 2023). Thus, cerebrovascular deficits with ageing arise from dysfunction of the cells of the neurovascular unit, including endothelial cells, pericytes and astrocytes (Figure 1).

## 4 Molecular drivers of cerebrovascular ageing

Oxidative stress and inflammatory signalling are prominent mechanisms leading to the age‐related impairments in cerebrovascular cells. These mechanisms are triggered by both extrinsic factors, such as those described below (extracranial vascular factors), and through intrinsic ageing processes. While not described in this review, many of the ‘hallmarks of ageing’ (Donato et al., 2015; López‐Otín et al., 2023), such as genomic instability, loss of proteostasis and deregulated nutrient signalling, occur within vascular cells with ageing. Specific to the brain, even healthy ageing is associated with an increase in Aβ and hyperphosphorylated tau that impact vascular function (Figure 1).

### 4.1 Oxidative stress and inflammatory signalling

Oxidative stress results from an imbalance between reactive oxygen species (ROS) production and antioxidant defences. Key contributors to increased vascular ROS with ageing are NADPH oxidase, the mitochondrial electron transport system, and uncoupled eNOS (Donato et al., 2015). Endothelial cells in the BBB have a higher content of mitochondria when compared to other capillary beds (Oldendorf et al., 1977). As such, mitochondrial ROS production could be particularly relevant to the cerebral vasculature. The brain is extremely vulnerable to oxidative damage due to its relatively high amount of lipids that are easily oxidized and lacks a strong antioxidant defence (Markesbery, 1999). Furthermore, protein oxidation in the brain is known to be particularly detrimental, as it can result in the formation of advanced glycation end‐products (AGEs) in the presence of monosaccharides (Smith et al., 1994; Huang et al., 2016), a potential contributor to arterial stiffening. Specific to the vasculature, superoxide reacts with NO, thereby reducing NO bioavailability and vasodilatory signalling (Donato et al., 2015). However, antioxidant supplementation does not treat cognitive decline in LOAD (Persson et al., 2014); thus, effective targeting of cerebrovascular oxidative stress remains a gap in the field.

Oxidative stress increases in tandem with inflammatory signalling. Indeed, advancing age is a state of chronic low‐grade inflammation (Franceschi et al., 2018). Age‐related increases in inflammatory signalling, particularly via nuclear factor κB (NF‐κB), lead to endothelial cell dysfunction (Walker et al., 2014). In the brain, activated microglia and the subsequent release of pro‐inflammatory cytokines contribute to neuroinflammation (Ferreira et al., 2014). Microglia are involved in Aβ clearance in the brain, and in response to Aβ release, pro‐inflammatory cytokines lead to the recruitment of additional microglia. Studies with AD mouse models have shown microglia to become less efficient in Aβ clearance, accompanied by decreased microglial phagocytic activity and increased Aβ plaque burden (Krabbe et al., 2013). In summary, both oxidative stress and inflammatory signalling are increased with advancing age and are also key to the progression of LOAD.

### 4.2 Amyloid‐β and tau

The most prominent theory for the progression of AD is the Aβ hypothesis, specifically that Aβ triggers tau pathology and neuronal toxicity; however, Aβ is also present in the brain of healthy older adults without clinical disease (Kapasi et al., 2017). While the effects of Aβ on neurons have been extensively studied, the effects of Aβ on the vasculature are less well known but may be equally important for the onset and progression of LOAD. The cleavage of amyloid precursor protein (APP) leads to the formation of Aβ peptides of different sizes depending on the cleavage sites. Aβ_1–40_ has the highest concentration in the brains of healthy individuals and LOAD patients, while the less prevalent Aβ_1–42_ is thought to be more pathogenic by stimulating aggregation. The excess cleavage of APP contributes to vascular dysfunction both by a loss of functional full‐length APP and by the production of toxic Aβ peptides. The knockdown of endothelial APP leads to impaired endothelium‐dependent vasodilation due to lower NO bioavailability in cerebral arteries from mice and downregulated eNOS expression in human brain microvascular endothelial cells (d'Uscio et al., 2018). At the same time, toxic Aβ_1–42_ induces capillary vasoconstriction by stimulating endothelin‐1 signalling (Nortley et al., 2019). Aβ also stimulates ROS generation, further impairing endothelium‐dependent vasodilation (Kadowaki et al., 2005). Mice with mutations in APP that lead to excessive Aβ production have impaired CBF, endothelial cell function and cerebral autoregulation, and these impairments occur before the appearance of Aβ plaques (Zhang et al., 1997). A separate condition that often co‐occurs with AD is cerebral amyloid angiopathy (CAA). CAA is characterized by a buildup of Aβ within the walls of the cerebral arterioles and an increased risk for haemorrhage and cognitive decline (Swarup et al., 2023).

Independent of Aβ, tau proteinopathy also induces vascular damage. Tau proteins stabilize microtubules in neurons and are needed for proper neuronal function. In LOAD, tau becomes hyperphosphorylated, leading to the aggregation and formation of fibrils. Tau protofibrils and fibrils activate pro‐inflammatory signalling in endothelial cells and disrupt barrier function (Guzmán‐Hernández & Fossati, 2025). Tau aggregates in brain microvascular endothelial cells also lead to a significant reduction of eNOS translocation to the cell membrane, causing reduced NO production (Hussong et al., 2023). Tau and neurofibrillary tangles accumulate around the cerebral vasculature in the brains of CAA‐positive AD patients (Hoglund et al., 2024). The buildup of tau around cortical arterioles is associated with smaller vessel diameters, shorter vessel lengths and lower blood vessel density (Bennett et al., 2018). Together, the accumulation of Aβ and the hyperphosphorylation of tau are present with advancing age and are key contributors to cerebrovascular dysfunction with LOAD.

## 5 Influence of extracranial vascular function on cerebrovascular ageing

The cerebrovascular cell impairments that arise with advancing age have been linked with age‐related changes to extracranial arteries. In particular, increases in blood pressure and arterial stiffness result in mechanical stresses to the cerebral vasculature, while atherosclerosis can impede blood flow from entering the brain.

### 5.1 Blood pressure

High blood pressure is one of the most significant risk factors for LOAD and cognitive impairment. It is not just high blood pressure in old age that is a LOAD risk, but high blood pressure in midlife, decades before the onset of LOAD symptoms, and the risk remains even if hypertension is treated later in life (Launer et al., 1995). High blood pressure is associated with lower CBF and impaired NVC in healthy older adults (Tryambake et al., 2013; Lefferts et al., 2018). Blood pressure puts tangential stress on the blood vessel wall, and when blood pressure is high, the wall adapts to the increased stress. This response includes a thickening of the walls of arteries and arterioles along with collagen deposition, ultimately leading to an increase in vascular stiffness (Pires et al., 2013). Within the carotid artery, this stiffening results in the resetting of the baroreceptor and decreased sensitivity, ultimately failing to produce effective blood pressure regulation (Mattace‐Raso et al., 2007). Within the brain, autoregulation maintains stable blood flow in the presence of unstable blood and perfusion pressure. While hypertension is associated with impaired autoregulation (Toth et al., 2014), the findings for the impact of primary ageing on autoregulation are inconsistent (Carey et al., 2000; Heckmann et al., 2003). Thus, while older age is associated with high blood pressure, it is not clear that ageing per se is associated with impaired autoregulation. Contributing to the age‐related increases in blood pressure is the renin–angiotensin–aldosterone system (RAAS). Chronic activation of the RAAS, both systemically and in the brain, leads to an increase in neuroinflammation, an increase in oxidative stress, an increase in BBB permeability, and a decrease in CBF (Mogi et al., 2012; Cifuentes et al., 2015). As such, there are many avenues by which high blood pressure may contribute to LOAD, including via increases in arterial stiffness.

### 5.2 Arterial stiffness

A common characteristic of advancing age is the changes in vascular structure that lead to stiffening of the arteries. These structural changes include increased elastin fragmentation, the formation of AGEs that contribute to collagen crosslink formation, and increased vascular smooth muscle cell tone (Lakatta & Levy, 2003). The large arteries, such as the carotids and the aorta, are of particular interest in the progression of arterial stiffness. Young, healthy and compliant large arteries are capable of effectively damping pulse pressure. As large arteries stiffen with age, this ability to damp pulse pressure decreases, resulting in higher pulse pressure being transmitted into the microcirculation (Mitchell, 2018). Organs with high blood flow and low resistance, such as the brain, are particularly vulnerable to this undamped pulsatile pressure (Waldstein et al., 2008).

Increased arterial stiffness and pulse pressure are associated with a plethora of issues, including reduced CBF, neuroinflammation and cognitive decline (Waldstein et al., 2008; Mitchell, 2018). Indeed, a meta‐analysis of 29 cross sectional and nine longitudinal studies found negative correlations between arterial stiffness and the cognitive domains of executive function and memory (Alvarez‐Bueno et al., 2020). Strikingly, aortic stiffness was predictive of the conversion from mild cognitive impairment to dementia in males and females, aged 55–98 years (Rouch et al., 2018). Furthermore, arterial stiffness and pulse pressure are also closely associated with increased Aβ deposition in AD patients (Hughes et al., 2018). In addition, the increases in pulse pressure with ageing could lead to a stiffening of the cerebral arterioles. These age‐related reductions in arteriole distensibility are potential contributors to impaired glymphatic function (Kress et al., 2014; Li et al., 2021). Thus, averting the age‐related increases in arterial stiffness is a potential target for preventing LOAD.

### 5.3 Atherosclerosis

The development of atherosclerosis is an age‐related process rooted in inflammatory signalling and endothelial dysfunction (Ross, 1999; Lakatta & Levy, 2003). Atherosclerosis in the carotid artery, as indicated by carotid plaques or greater intima–media thickness (IMT), is more prevalent in patients with dementia; however, this association appears to be stronger for VCI than for AD (Carcaillon et al., 2015; Gustavsson et al., 2020). Furthermore, in patients without clinical dementia, carotid IMT is related to poor memory performance (Romero et al., 2009). Interestingly, peripheral artery disease is also related to the risk for AD (Dearborn et al., 2017). The mechanisms underlying these associations are not entirely clear but may be due to reduced cerebral perfusion, as seen with carotid artery stenosis, or to shared risk factors, such as hypertension, endothelial dysfunction, and the *APOE4* genotype (Carcaillon et al., 2015; Dearborn et al., 2017). Moreover, extracranial atherosclerosis may reflect the presence of intracranial atherosclerosis and microvascular dysfunction, which affect blood flow and the brain's inflammatory environment. Intracranial atherosclerosis and related infarcts are key features of VCI (Dearborn et al., 2017; Iadecola et al., 2019). In summary, there are strong associations between atherosclerosis and VCI, and as many patients have a mixed dementia that comprises both AD and VCI, the contributions of atherosclerosis are a key feature of the age‐related declines.

## 6 Integration of cerebrovascular deficits with systemic, cellular, and molecular mechanisms

**FIGURE 2:**
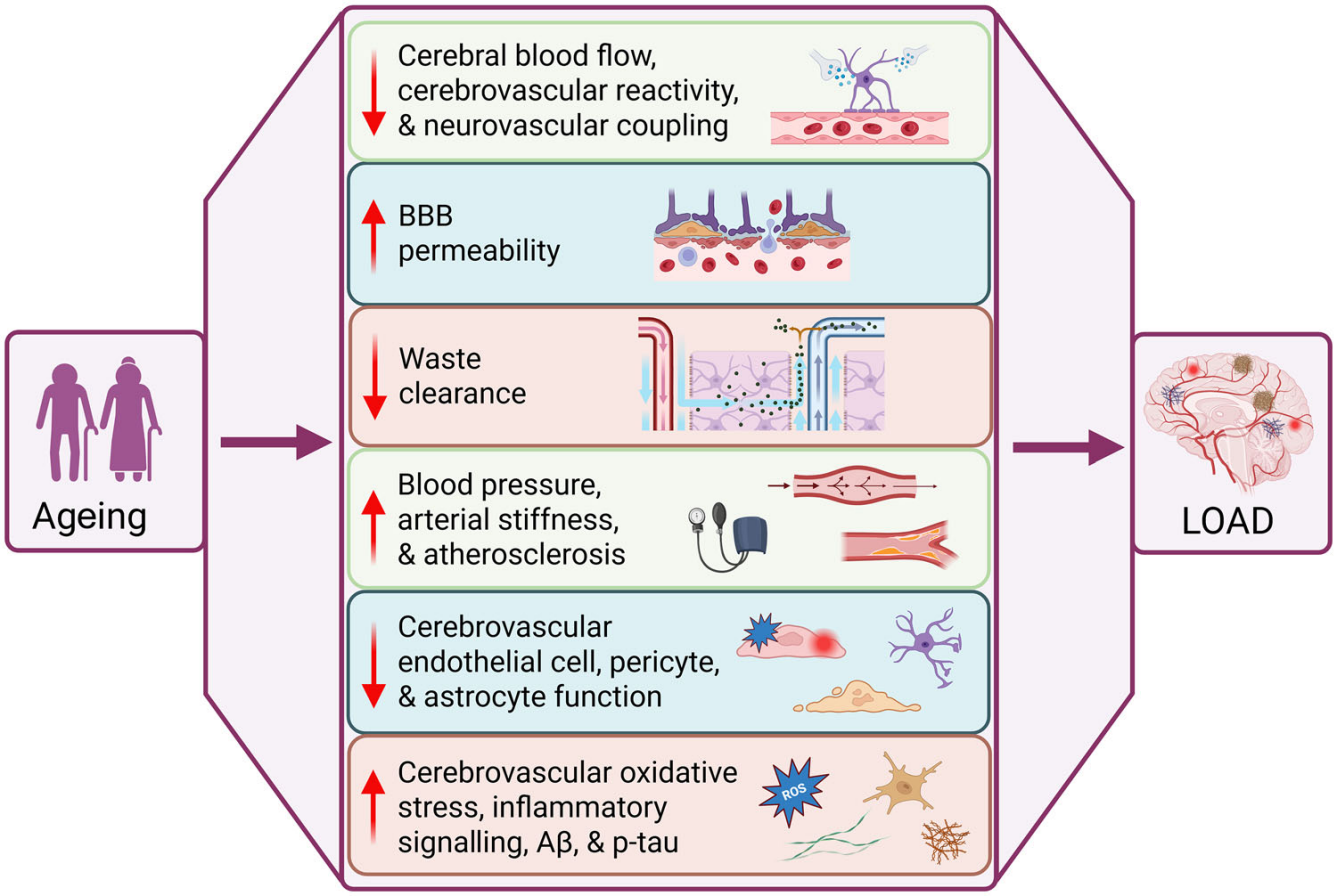
Contributions of vascular ageing to late‐onset Alzheimer's disease (LOAD). Advancing age is associated with cerebrovascular deficits, such as reduced cerebral blood flow, cerebrovascular reactivity and neurovascular coupling, as well as increased blood–brain barrier (BBB) permeability and impaired waste clearance. The initiating factors for these deficits could arise from age‐related increases in blood pressure, arterial stiffness and atherosclerosis. Moreover, impairment to the cells of the neurovascular unit contributes to these deficits, which are triggered by molecular mechanisms such as increased oxidative stress, inflammatory signalling, amyloid‐β (Aβ) and hyperphosphorylated tau (p‐tau). Image created with BioRender.

A healthy vasculature serves many purposes for the brain, with a primary role in delivering nutrients and other substances, as well as removing waste. CBF accomplishes the goal of delivery, with the precision added by healthy CVR and NVC, as well as a functional BBB. The removal of waste occurs through the blood as well as the glymphatic system, both of which rely on healthy cerebrovascular function. Age‐related impairments in CBF, CVR, NVC, BBB integrity and glymphatics are recognized as potential contributors to LOAD, with their causes arising from extracranial, cellular and molecular sources (Figure 2). For example, high blood pressure and stiffer large elastic arteries, two key features of vascular ageing, create mechanical forces that damage the cerebral vasculature. At the cellular level, cells of the neurovascular unit contribute to the regulation of blood flow and permeability. Age‐related dysfunction of endothelial cells, pericytes, and astrocytes leads to poor CVR and NVC and a leakier BBB. The ageing of vascular cells is driven by both intrinsic processes and external influences, including molecular mechanisms of ageing and negative forces arising from extracranial vascular ageing. A key feature of vascular ageing is an increase in oxidative stress and inflammatory signalling, leading to impaired endothelial function and stiffening of the extracellular matrix. With advancing age in general, but particularly in the context of LOAD, there is an increase in Aβ and hyperphosphorylated tau, which further contribute to the pro‐inflammatory and pro‐oxidant environment in the cerebral vasculature. From functional outcomes to extracranial influences to cellular and molecular mechanisms, there is a high degree of interconnection, feedback, and vicious cycles.

While it is possible to conceptualize a linear pathway starting with age‐related increases in arterial stiffness and blood pressure, leading to cerebrovascular cellular and molecular mechanisms of dysfunction, and ultimately impairing CBF, NVC and BBB integrity, this representation does not accurately reflect the complexity of the underlying physiology. For example, endothelial cell dysfunction can impair NVC and cause tissue hypoxia, triggering a pro‐inflammatory environment and a subsequent increase in Aβ production, which further impairs endothelial function. Similarly, high blood pressure triggers an adaptive response in arteries that stiffens them, and these stiffer arteries further amplify pulse pressure, contributing to higher systolic pressure, particularly in downstream blood vessels. Furthermore, molecular mechanisms such as increased oxidative stress and inflammatory signalling will have direct impacts throughout the vascular tree, from the cells in extracranial arteries to larger cerebral arteries to the microvasculature. As such, the current treatment approach of targeting a single mechanism, such as antihypertensive or anti‐Aβ antibody treatments, will have a limited impact. In contrast, targeting the root causes of age‐related vascular dysfunction could be a more effective approach to treating LOAD.

## 7 Conclusions

LOAD is a complex neurodegenerative disease driven by pathophysiology across multiple cell types and organ systems. Numerous lines of evidence support the strong association between vascular dysfunction and the progression of LOAD. Vascular dysfunction in the brain influences not only blood flow but also waste clearance. Cerebrovascular ageing is preceded by stiffening of the large arteries and elevated blood pressure and is associated with the accumulation of harmful substances, including Aβ, hyperphosphorylated tau, ROS and inflammatory mediators. By targeting these mechanisms, interventions that counteract vascular ageing have the potential to prevent the onset of LOAD.

## AUTHOR CONTRIBUTIONS

Skylyn J. Ferguson, Young D. Choi and Ashley E. Walker contributed to conceptualizing, drafting, and editing the manuscript. All authors have read and approved the final version of this manuscript and agree to be accountable for all aspects of the work in ensuring that questions related to the accuracy or integrity of any part of the work are appropriately investigated and resolved. All persons designated as authors qualify for authorship, and all those who qualify for authorship are listed.

## CONFLICT OF INTEREST

None declared.

## References

[eph70288-bib-0001] Alvarez‐Bueno, C. , Cunha, P. G , Martinez‐Vizcaino, V. , Pozuelo‐Carrascosa, D. P. , Visier‐Alfonso, M. E. , Jimenez‐Lopez, E. , & Cavero‐Redondo, I. (2020). Arterial stiffness and cognition among adults: A systematic review and meta‐analysis of observational and longitudinal studies. Journal of the American Heart Association, 9, e014621. 32106748 10.1161/JAHA.119.014621 PMC7335587

[eph70288-bib-0002] Alwatban, M. , Murman, D. L. , & Bashford, G. (2019). Cerebrovascular reactivity impairment in preclinical Alzheimer's disease. Journal of Neuroimaging, 29, 493–498. 30748053 10.1111/jon.12606

[eph70288-bib-0003] Bangen, K. J. , Clark, A. L. , Edmonds, E. C. , Evangelista, N. D. , Werhane, M. L. , Thomas, K. R. , Locano, L. E. , Tran, M. , Zlatar, Z. Z. , Nation, D. A. , Bondi, M. W. , & Delano‐Wood, L. (2017). Cerebral blood flow and amyloid‐β interact to affect memory performance in cognitively normal older adults. Frontiers in Aging Neuroscience, 9, 181. 28642699 10.3389/fnagi.2017.00181 PMC5463038

[eph70288-bib-0004] Bell, R. D. , Winkler, E. A. , Sagare, A. P. , Singh, I. , LaRue, B. , Deane, R. , & Zlokovic, B. V. (2010). Pericytes control key neurovascular functions and neuronal phenotype in the adult brain and during brain aging. Neuron, 68, 409–427. 21040844 10.1016/j.neuron.2010.09.043 PMC3056408

[eph70288-bib-0005] Bennett, R. E. , Robbins, A. B. , Hu, M. , Cao, X. , Betensky, R. A. , Clark, T. , Das, S. , & Hyman, B. T. (2018). Tau induces blood vessel abnormalities and angiogenesis‐related gene expression in P301L transgenic mice and human Alzheimer's disease. Proceedings of the National Academy of Sciences of the United States of America, 115, E1289–E1298. 29358399 10.1073/pnas.1710329115 PMC5819390

[eph70288-bib-0006] Cantin, S. , Villien, M. , Moreaud, O. , Tropres, I. , Keignart, S. , Chipon, E. , Le Bas, J.‐F. , Warnking, J. , & Krainik, A. (2011). Impaired cerebral vasoreactivity to CO_2_ in Alzheimer's disease using BOLD fMRI. Neuroimage, 58, 579–587. 21745581 10.1016/j.neuroimage.2011.06.070

[eph70288-bib-0007] Carcaillon, L. , Plichart, M. , Zureik, M. , Rouaud, O. , Majed, B. , Ritchie, K. , Tzourio, C. , Dartigues, J.‐F. , & Empana, J.‐P. (2015). Carotid plaque as a predictor of dementia in older adults: The Three‐City Study. Alzheimer's & Dementia, 11, 239–248. 10.1016/j.jalz.2014.07.160 25510384

[eph70288-bib-0008] Carey, B. J. , Eames, P. J. , Blake, M. J. , Panerai, R. B. , & Potter, J. F. (2000). Dynamic cerebral autoregulation is unaffected by aging. Stroke, 31, 2895–2900. 11108745 10.1161/01.str.31.12.2895

[eph70288-bib-0009] Cifuentes, D. , Poittevin, M. , Dere, E. , Broquères‐You, D. , Bonnin, P. , Benessiano, J. , Pocard, M. , Mariani, J. , Kubis, N. , Merkulova‐Rainon, T. , & Lévy, B. I. (2015). Hypertension accelerates the progression of Alzheimer‐like pathology in a mouse model of the disease. Hypertension, 65, 218–224. 25331846 10.1161/HYPERTENSIONAHA.114.04139

[eph70288-bib-0010] Clarke, L. E. , Liddelow, S. A. , Chakraborty, C. , Münch, A. E. , Heiman, M. , & Barres, B. A. (2018). Normal aging induces A1‐like astrocyte reactivity. Proceedings of the National Academy of Sciences of the United States of America, 115, E1896–E1905. 29437957 10.1073/pnas.1800165115 PMC5828643

[eph70288-bib-0011] Dagum, P. , Elbert, D. L , Giovangrandi, L. , Singh, T. , Venkatesh, V. V , Corbellini, A. , Kaplan, R. M. , Levendovszky, S. R. , Ludington, E. , Yarasheski, K. , Lowenkron, J. , VandeWeerd, C. , Lim, M. M. , & Iliff, J. J. (2026). The glymphatic system clears amyloid beta and tau from brain to plasma in humans. Nature Communications, 17, 715. 10.1038/s41467-026-68374-8 PMC12847902 41593094

[eph70288-bib-0012] Dearborn, J. L , Zhang, Y. , Qiao, Y. , Suri, M. F. K. , Liu, L. , Gottesman, R. F. , Rawlings, A. M. , Mosley, T. H. , Alonso, A. , Knopman, D. S. , Guallar, E. , & Wasserman, B. A. (2017). Intracranial atherosclerosis and dementia. Neurology, 88, 1556–1563. 28330958 10.1212/WNL.0000000000003837 PMC5395073

[eph70288-bib-0013] Donato, A. J. , Morgan, R. G. , Walker, A. E. , & Lesniewski, L. A. (2015). Cellular and molecular biology of aging endothelial cells. Journal of Molecular and Cellular Cardiology, 89, 122–135. 25655936 10.1016/j.yjmcc.2015.01.021 PMC4522407

[eph70288-bib-0014] d'Uscio, L. V. , He, T. , Santhanam, A. V. , & Katusic, Z. S. (2018). Endothelium‐specific amyloid precursor protein deficiency causes endothelial dysfunction in cerebral arteries. Journal of Cerebral Blood Flow and Metabolism, 38, 1715–1726. 28959912 10.1177/0271678X17735418 PMC6168907

[eph70288-bib-0015] Fazlollahi, A. , Calamante, F. , Liang, X. , Bourgeat, P. , Raniga, P. , Dore, V. , Fripp, J. , Ames, D. , Masters, C. L. , Rowe, C. C. , Connelly, A. , Villemagne, V. L , & Salvado, O. , & Australian Imaging Biomarkers and Lifestyle (AIBL) Research Group . (2020). Increased cerebral blood flow with increased amyloid burden in the preclinical phase of alzheimer's disease. Journal of Magnetic Resonance Imaging, 51, 505–513. 31145515 10.1002/jmri.26810

[eph70288-bib-0016] Ferreira, S. T. , Clarke, J. R. , Bomfim, T. R. , & De Felice, F. G. (2014). Inflammation, defective insulin signaling, and neuronal dysfunction in Alzheimer's disease. Alzheimer's & Dementia, 10, S76–S83. 10.1016/j.jalz.2013.12.010 24529528

[eph70288-bib-0017] Franceschi, C. , Garagnani, P. , Parini, P. , Giuliani, C. , & Santoro, A. (2018). Inflammaging: A new immune‐metabolic viewpoint for age‐related diseases. Nature Reviews Endocrinology, 14, 576–590. 10.1038/s41574-018-0059-4 30046148

[eph70288-bib-0018] Gorelick, P. B. , Scuteri, A. , Black, S. E. , Decarli, C. , Greenberg, S. M. , Iadecola, C. , Launer, L. J. , Laurent, S. , Lopez, O. L. , Nyenhuis, D. , Petersen, R. C. , Schneider, J. A. , Tzourio, C. , Arnett, D. K. , Bennett, D. A. , Chui, H. C. , Higashida, R. T. , Lindquist, R. , Nilsson, P. M. , Roman, G. C. , … American Heart Association Stroke Council, Council on Epidemiology and Prevention, Council on Cardiovascular Nursing, Council on Cardiovascular Radiology and Intervention, and Council on Cardiovascular Surgery and Anesthesia . (2011). Vascular contributions to cognitive impairment and dementia: A statement for healthcare professionals from the american heart association/american stroke association. Stroke, 42, 2672–2713. 21778438 10.1161/STR.0b013e3182299496 PMC3778669

[eph70288-bib-0019] Gustavsson, A. , van Westen, D. , Stomrud, E. , Engström, G. , Nägga, K. , & Hansson, O. (2020). Midlife atherosclerosis and development of Alzheimer or vascular dementia. Annals of Neurology, 87, 52–62. 31721283 10.1002/ana.25645 PMC6973178

[eph70288-bib-0020] Guzmán‐Hernández, R. , & Fossati, S. (2025). Fibrillar tau alters cerebral endothelial cell metabolism, vascular inflammatory activation, and barrier function in vitro and in vivo. Alzheimer's & Dementia, 21, e70077. 10.1002/alz.70077 PMC11923556 40110691

[eph70288-bib-0021] Heckmann, J. G. , Brown, C. M. , Cheregi, M. , Hilz, M. J. , & Neundörfer, B. (2003). Delayed cerebrovascular autoregulatory response to ergometer exercise in normotensive elderly humans. Cerebrovascular Diseases, 16, 423–429. 13130185 10.1159/000072567

[eph70288-bib-0022] Hoglund, Z. , Ruiz‐Uribe, N. , del Sastre, E. , Woost, B. , Bader, E. , Bailey, J. , Hyman, B. T. , Zwang, T. , & Bennett, R. E. (2024). Brain vasculature accumulates tau and is spatially related to tau tangle pathology in Alzheimer's disease. Acta Neuropathologica, 147, 101. 38884806 10.1007/s00401-024-02751-9 PMC11182845

[eph70288-bib-0023] Huang, W.‐J. , Zhang, X. , & Chen, W.‐W. (2016). Role of oxidative stress in Alzheimer's disease. Biomedical Reports, 4, 519–522. 27123241 10.3892/br.2016.630 PMC4840676

[eph70288-bib-0024] Hughes, T. M. , Wagenknecht, L. E. , Craft, S. , Mintz, A. , Heiss, G. , Palta, P. , Wong, D. , Zhou, Y. , Knopman, D. , Mosley, T. H. , & Gottesman, R. F. (2018). Arterial stiffness and dementia pathology. Neurology, 90, e1248–e1256. 29549223 10.1212/WNL.0000000000005259 PMC5890613

[eph70288-bib-0025] Hussong, S. A. , Banh, A. Q. , Van Skike, C. E. , Dorigatti, A. O. , Hernandez, S. F. , Hart, M. J. , Ferran, B. , Makhlouf, H. , Gaczynska, M. , Osmulski, P. A. , McAllen, S. A. , Dineley, K. T. , Ungvari, Z. , Perez, V. I. , Kayed, R. , & Galvan, V. (2023). Soluble pathogenic tau enters brain vascular endothelial cells and drives cellular senescence and brain microvascular dysfunction in a mouse model of tauopathy. Nature Communications, 14, 2367. 10.1038/s41467-023-37840-y PMC10126555 37185259

[eph70288-bib-0026] Iadecola, C. , Duering, M. , Hachinski, V. , Joutel, A. , Pendlebury, S. T. , Schneider, J. A. , & Dichgans, M. (2019). Vascular cognitive impairment and dementia: JACC scientific expert panel. Journal of the American College of Cardiology, 73, 3326–3344. 31248555 10.1016/j.jacc.2019.04.034 PMC6719789

[eph70288-bib-0027] Iadecola, C. , & Gottesman, R. F. (2018). Cerebrovascular alterations in Alzheimer's disease: Incidental or pathogenic? Circulation Research, 123, 406–408. 30355253 10.1161/CIRCRESAHA.118.313400 PMC6214471

[eph70288-bib-0028] Iliff, J. J , Wang, M. , Liao, Y. , Plogg, B. A , Peng, W. , Gundersen, G. A , Benveniste, H. , Vates, G. E. , Deane, R. , Goldman, S. A. , Nagelhus, E. A. , & Nedergaard, M. (2012). A paravascular pathway facilitates CSF flow through the brain parenchyma and the clearance of interstitial solutes, including amyloid β. Science Translational Medicine, 4, 147ra111. 10.1126/scitranslmed.3003748 PMC3551275 22896675

[eph70288-bib-0029] Iturria‐Medina, Y. , Sotero, R. C. , Toussaint, P. J. , Mateos‐Pérez, J. M. , & Evans, A. C. (2016). Early role of vascular dysregulation on late‐onset Alzheimer's disease based on multifactorial data‐driven analysis. Nature Communications, 7, 11934. 10.1038/ncomms11934 PMC4919512 27327500

[eph70288-bib-0030] Kadowaki, H. , Nishitoh, H. , Urano, F. , Sadamitsu, C. , Matsuzawa, A. , Takeda, K. , Masutani, H. , Yodoi, J. , Urano, Y. , Nagano, T. , & Ichijo, H. (2005). Amyloid β induces neuronal cell death through ROS‐mediated ASK1 activation. Cell Death and Differentiation, 12, 19–24. 15592360 10.1038/sj.cdd.4401528

[eph70288-bib-0031] Kapasi, A. , DeCarli, C. , & Schneider, J. A. (2017). Impact of multiple pathologies on the threshold for clinically overt dementia. Acta Neuropathologica, 134, 171–186. 28488154 10.1007/s00401-017-1717-7 PMC5663642

[eph70288-bib-0032] Krabbe, G. , Halle, A. , Matyash, V. , Rinnenthal, J. L , Eom, G. D. , Bernhardt, U. , Miller, K. R. , Prokop, S. , Kettenmann, H. , & Heppner, F. L. (2013). Functional impairment of microglia coincides with beta‐amyloid deposition in mice with Alzheimer‐like pathology. PLoS ONE, 8, e60921. 23577177 10.1371/journal.pone.0060921 PMC3620049

[eph70288-bib-0033] Kress, B. T. , Iliff, J. J. , Xia, M. , Wang, M. , Wei, H. S. , Zeppenfeld, D. , Xie, L. , Kang, H. , Xu, Q. , Liew, J. A. , Plog, B. A. , Ding, F. , Deane, R. , & Nedergaard, M. (2014). Impairment of paravascular clearance pathways in the aging brain. Annals of Neurology, 76, 845–861. 25204284 10.1002/ana.24271 PMC4245362

[eph70288-bib-0034] Lakatta, E. G. , & Levy, D. (2003). Arterial and cardiac aging: Major shareholders in cardiovascular disease enterprises: Part I: Aging arteries: A “set up” for vascular disease. Circulation, 107, 139–146. 12515756 10.1161/01.cir.0000048892.83521.58

[eph70288-bib-0035] Launer, L. J. , Masaki, K. , Petrovitch, H. , Foley, D. , & Havlik, R. J. (1995). The association between midlife blood pressure levels and late‐life cognitive function. The Honolulu‐Asia Aging Study. The Journal of the American Medical Association, 274, 1846–1851. 7500533

[eph70288-bib-0036] Lefferts, W. K. , DeBlois, J. P. , Barreira, T. V. , & Heffernan, K. S. (2018). Neurovascular coupling during cognitive activity in adults with controlled hypertension. Journal of Applied Physiology, 125, 1906–1916. 30048202 10.1152/japplphysiol.00100.2018

[eph70288-bib-0037] Li, M. , Kitamura, A. , Beverley, J. , Koudelka, J. , Duncombe, J. , Lennen, R. , Jansen, M. A. , Marshall, I. , Platt, B. , Wiegand, U. K. , Carare, R. O. , Kalaria, R. N. , Iliff, J. J. , & Horsburgh, K. (2021). Impaired glymphatic function and pulsation alterations in a mouse model of vascular cognitive impairment. Frontiers in Aging Neuroscience, 13, 788519. 35095472 10.3389/fnagi.2021.788519 PMC8793139

[eph70288-bib-0038] López‐Otín, C. , Blasco, M. A , Partridge, L. , Serrano, M. , & Kroemer, G. (2023). Hallmarks of aging: An expanding universe. Cell, 186, 243–278. 36599349 10.1016/j.cell.2022.11.001

[eph70288-bib-0039] Lourenço, C. F. , Ledo, A. , Barbosa, R. M. , & Laranjinha, J. (2017). Neurovascular uncoupling in the triple transgenic model of Alzheimer's disease: Impaired cerebral blood flow response to neuronal‐derived nitric oxide signaling. Experimental Neurology, 291, 36–43. 28161255 10.1016/j.expneurol.2017.01.013

[eph70288-bib-0040] Lu, H. , Xu, F. , Rodrigue, K. M. , Kennedy, K. M. , Cheng, Y. , Flicker, B. , Hebrank, A. C. , Uh, J. , & Park, D. C. (2011). Alterations in cerebral metabolic rate and blood supply across the adult lifespan. Cerebral Cortex, 21, 1426–1434. 21051551 10.1093/cercor/bhq224 PMC3097991

[eph70288-bib-0041] Markesbery, W. R. (1999). The role of oxidative stress in Alzheimer disease. Archives of Neurology, 56, 1449–1452. 10593298 10.1001/archneur.56.12.1449

[eph70288-bib-0042] Masters, C. L , Bateman, R. , Blennow, K. , Rowe, C. C. , Sperling, R. A. , & Cummings, J. L. (2015). Alzheimer's disease. Nature Reviews. Disease Primers, 1, 15056. 10.1038/nrdp.2015.56 27188934

[eph70288-bib-0043] Mattace‐Raso, F. U. , Van Den Meiracker, A. H. , Bos, W. J. , Van Der Cammen, T. J. , Westerhof, B. E. , Elias‐Smale, S. , Reneman, R. S. , Hoeks, A. P. , Hofman, A. , & Witteman, J. C. (2007). Arterial stiffness, cardiovagal baroreflex sensitivity and postural blood pressure changes in older adults: The Rotterdam Study. Journal of Hypertension, 25, 1421–1426. 17563564 10.1097/HJH.0b013e32811d6a07

[eph70288-bib-0044] Mitchell, G. F. (2018). Aortic stiffness, pressure and flow pulsatility, and target organ damage. Journal of Applied Physiology, 125, 1871–1880. 30359540 10.1152/japplphysiol.00108.2018 PMC6842890

[eph70288-bib-0045] Mogi, M. , Iwanami, J. , & Horiuchi, M. (2012). Roles of brain angiotensin II in cognitive function and dementia. International Journal of Hypertension, 2012, 169649. 23304450 10.1155/2012/169649 PMC3529904

[eph70288-bib-0046] Montagne, A. , Barnes, S. R. , Sweeney, M. D. , Halliday, M. R. , Sagare, A. P. , Zhao, Z. , Toga, A. W. , Jacobs, R. E. , Liu, C. Y. , Amezcua, L. , Harrington, M. G. , Chui, H. C. , Law, M. , & Zlokovic, B. V. (2015). Blood‐brain barrier breakdown in the aging human hippocampus. Neuron, 85, 296–302. 25611508 10.1016/j.neuron.2014.12.032 PMC4350773

[eph70288-bib-0047] Mukli, P. , Pinto, C. B. , Owens, C. D. , Csipo, T. , Lipecz, A. , Szarvas, Z. , Peterfi, A. , Langley, A. C. D. C. P. , Hoffmeister, J. , Racz, F. S. , Perry, J. W. , Tarantini, S. , Nyúl‐Tóth, Á. , Sorond, F. A. , Yang, Y. , James, J. A. , Kirkpatrick, A. C. , Prodan, C. I. , Toth, P. , … Yabluchanskiy, A. (2023). Impaired neurovascular coupling and increased functional connectivity in the frontal cortex predict age‐related cognitive dysfunction. Advancement of Science, 11, 2303516. 10.1002/advs.202303516 PMC10962492 38155460

[eph70288-bib-0048] Nortley, R. , Korte, N. , Izquierdo, P. , Hirunpattarasilp, C. , Mishra, A. , Jaunmuktane, Z. , Kyrargyri, V. , Pfeiffer, T. , Khennouf, L. , Madry, C. , Gong, H. , Richard‐Loendt, A. , Huang, W. , Saito, T. , Saido, T. C , Brandner, S. , Sethi, H. , & Attwell, D. (2019). Amyloid β oligomers constrict human capillaries in Alzheimer's disease via signaling to pericytes. Science, 365, eaav9518. 31221773 10.1126/science.aav9518. PMC6658218

[eph70288-bib-0049] Oldendorf, W. H , Cornford, M. E , & & Brown, W. J. (1977). The large apparent work capability of the blood‐brain barrier: A study of the mitochondrial content of capillary endothelial cells in brain and other tissues of the rat. Annals of Neurology, 1, 409–417. 617259 10.1002/ana.410010502

[eph70288-bib-0050] Pardo, K. , Khasminsky, V. , Keret, O. , Benninger, F. , Goldberg, I. , Shelef, I. , Auriel, E. , & Glik, A. (2025). Alzheimer's disease patients have smaller venous drainage system compared to cognitively healthy controls. Alzheimer's & Dementia, 21, e14551. 10.1002/alz.14551 PMC11851167 39936326

[eph70288-bib-0051] Peng, S.‐L. , Chen, X. , Li, Y. , Rodrigue, K. M. , Park, D. C. , & Lu, H. (2018). Age‐related changes in cerebrovascular reactivity and their relationship to cognition: A four‐year longitudinal study. Neuroimage, 174, 257–262. 29567504 10.1016/j.neuroimage.2018.03.033 PMC5949266

[eph70288-bib-0052] Persson, T. , Popescu, B. O. , & Cedazo‐Minguez, A. (2014). Oxidative stress in Alzheimer's disease: Why did antioxidant therapy fail? Oxidative Medicine and Cellular Longevity, 2014, 427318. 24669288 10.1155/2014/427318 PMC3941783

[eph70288-bib-0053] Pires, P. W. , Dams Ramos, C. M. , Matin, N. , & Dorrance, A. M. (2013). The effects of hypertension on the cerebral circulation. American Journal of Physiology‐Heart and Circulatory Physiology, 304, H1598–H1614. 23585139 10.1152/ajpheart.00490.2012 PMC4280158

[eph70288-bib-0054] Prohovnik, I. , Mayeux, R. , Sackeim, H. A. , Smith, G. , Stern, Y. , & Alderson, P. O. (1988). Cerebral perfusion as a diagnostic marker of early Alzheimer's disease. Neurology, 38, 931–937. 3368076 10.1212/wnl.38.6.931

[eph70288-bib-0055] Romero, J. R. , Beiser, A. , Seshadri, S. , Benjamin, E. J. , Polak, J. F. , Vasan, R. S. , Au, R. , DeCarli, C. , & Wolf, P. A. (2009). Carotid artery atherosclerosis, MRI indices of brain ischemia and aging and cognitive impairment: The Framingham Study. Stroke, 40, 1590–1596. 19265054 10.1161/STROKEAHA.108.535245 PMC2705324

[eph70288-bib-0056] Ross, R. (1999). Atherosclerosis—an inflammatory disease. New England Journal of Medicine, 340, 115–126. 9887164 10.1056/NEJM199901143400207

[eph70288-bib-0057] Rossman, M. J. , Kaplon, R. E. , Hill, S. D. , McNamara, M. N. , Santos‐Parker, J. R. , Pierce, G. L. , Seals, D. R. , & Donato, A. J. (2017). Endothelial cell senescence with aging in healthy humans: Prevention by habitual exercise and relation to vascular endothelial function. American Journal of Physiology‐Heart and Circulatory Physiology, 313, H890–H895. 28971843 10.1152/ajpheart.00416.2017 PMC5792201

[eph70288-bib-0058] Rouch, L. , Cestac, P. , Sallerin, B. , Andrieu, S. , Bailly, H. , Beunardeau, M. , Cohen, A. , Dubail, D. , Hernandorena, I. , Seux, M.‐L. , Vidal, J.‐S. , & Hanon, O. (2018). Pulse wave velocity is associated with greater risk of dementia in mild cognitive impairment patients. Hypertension, 72, 1109–1116. 30354804 10.1161/HYPERTENSIONAHA.118.11443

[eph70288-bib-0059] Rubanyi, G. M. , Romero, J. C. , & Vanhoutte, P. M. (1986). Flow‐induced release of endothelium‐derived relaxing factor. American Journal of Physiology, 250, H1145–H1149. 3487253 10.1152/ajpheart.1986.250.6.H1145

[eph70288-bib-0060] Ruitenberg, A. , den Heijer, T. , Bakker, S. L. M. , van Swieten, J. C. , Koudstaal, P. J. , Hofman, A. , & Breteler, M. M. B. (2005). Cerebral hypoperfusion and clinical onset of dementia: The Rotterdam Study. Annals of Neurology, 57, 789–794. 15929050 10.1002/ana.20493

[eph70288-bib-0061] Smith, M. A , Taneda, S. , Richey, P. L , Miyata, S. , Yan, S. D. , Stern, D. , Sayre, L. M. , Monnier, V. M. , & Perry, G. (1994). Advanced Maillard reaction end products are associated with Alzheimer disease pathology. Proceedings of the National Academy of Sciences of the United States of America, 91, 5710–5714. 8202552 10.1073/pnas.91.12.5710 PMC44066

[eph70288-bib-0062] Stackhouse, T. L , & Mishra, A. (2021). Neurovascular Coupling in Development and Disease: Focus on Astrocytes. Frontiers in Cell and Developmental Biology, 9, 702832. 34327206 10.3389/fcell.2021.702832 PMC8313501

[eph70288-bib-0063] Sur, S. , Lin, Z. , Li, Y. , Yasar, S. , Rosenberg, P. , Moghekar, A. , Hou, X. , Kalyani, R. , Hazel, K. , Pottanat, G. , Xu, C. , van Zijl, P. , Pillai, J. , Liu, P. , Albert, M. , & Lu, H. (2020). Association of cerebrovascular reactivity and Alzheimer pathologic markers with cognitive performance. Neurology, 95, e962–e972. 32661101 10.1212/WNL.0000000000010133 PMC7668551

[eph70288-bib-0064] Swarup, O. , Barker, J. L. , Watson, R. , Davis, S. M. , Campbell, B. C. V. , & Yassi, N. (2023). Cerebral amyloid angiopathy: Clinical presentations and management challenges in the Australian context. Internal Medicine Journal, 53, 907–916. 36565446 10.1111/imj.15999

[eph70288-bib-0065] Tarantini, S. , Tran, C. H. T. , Gordon, G. R. , Ungvari, Z. , & Csiszar, A. (2017). Impaired neurovascular coupling in aging and Alzheimer's disease: Contribution of astrocyte dysfunction and endothelial impairment to cognitive decline. Experimental Gerontology, 94, 52–58. 27845201 10.1016/j.exger.2016.11.004 PMC5429210

[eph70288-bib-0066] Tian, Y. , Fopiano, K. A. , Buncha, V. , Lang, L. , Suggs, H. A. , Wang, R. , Rudic, R. D. , Filosa, J. A. , & Bagi, Z. (2023). The role of ADAM17 in cerebrovascular and cognitive function in the APP/PS1 mouse model of Alzheimer's disease. Frontiers in Molecular Neuroscience, 16, 1125932. 36937050 10.3389/fnmol.2023.1125932 PMC10018024

[eph70288-bib-0067] Ting, K. K. , Coleman, P. , Kim, H. J. , Zhao, Y. , Mulangala, J. , Cheng, N. C. , Li, W. , Gunatilake, D. , Johnstone, D. M. , Loo, L. , Neely, G. G. , Yang, P. , Götz, J. , Vadas, M. A , & Gamble, J. R. (2023). Vascular senescence and leak are features of the early breakdown of the blood–brain barrier in Alzheimer's disease models. GeroScience, 45, 3307–3331. 37782439 10.1007/s11357-023-00927-x PMC10643714

[eph70288-bib-0068] Toth, P. , Tucsek, Z. , Tarantini, S. , Sosnowska, D. , Gautam, T. , Mitschelen, M. , Koller, A. , Sonntag, W. E. , Csiszar, A. , & Ungvari, Z. (2014). IGF‐1 deficiency impairs cerebral myogenic autoregulation in hypertensive mice. Journal of Cerebral Blood Flow and Metabolism, 34, 1887–1897. 25248835 10.1038/jcbfm.2014.156 PMC4269740

[eph70288-bib-0069] Tryambake, D. , He, J. , Firbank, M. J. , O'Brien, J. T , Blamire, A. M. , & Ford, G. A. (2013). Intensive blood pressure lowering increases cerebral blood flow in older subjects with hypertension. Hypertension, 61, 1309–1315. 23529166 10.1161/HYPERTENSIONAHA.112.200972

[eph70288-bib-0070] van Dijk, S. E. , Drenth, N. , Hafkemeijer, A. , Labadie, G. , Witjes‐Ané, M. W. , Baas, F. , Vreijling, J. P. , Blauw, G. J. , Rombouts, S. , van der Grond, J. , & van Rooden, S. (2025). Neurovascular decoupling is associated with lobar intracerebral hemorrhages and white matter hyperintensities. Journal of the American Heart Association, 14, e038819. 39950450 10.1161/JAHA.124.038819 PMC12074756

[eph70288-bib-0071] van Dinther, M. , Hooghiemstra, A. M. , Bron, E. E. , Versteeg, A. , Leeuwis, A. E. , Kalay, T. , Moonen, J. E. , Kuipers, S. , Backes, W. H. , Jansen, J. F. A. , van Osch, M. J. P. , Biessels, G.‐J. , Staals, J. , van Oostenbrugge, R. J. , & Consortium, H.‐B. C. (2024). Lower cerebral blood flow predicts cognitive decline in patients with vascular cognitive impairment. Alzheimer's & Dementia, 20, 136–144. 10.1002/alz.13408 PMC10917014 37491840

[eph70288-bib-0072] Waldstein, S. R. , Rice, S. C. , Thayer, J. F. , Najjar, S. S. , Scuteri, A. , & Zonderman, A. B. (2008). Pulse pressure and pulse wave velocity are related to cognitive decline in the Baltimore Longitudinal Study of Aging. Hypertension, 51, 99–104. 18025297 10.1161/HYPERTENSIONAHA.107.093674

[eph70288-bib-0073] Walker, A. E. , Kaplon, R. E. , Pierce, G. L. , Nowlan, M. J. , & Seals, D. R. (2014). Prevention of age‐related endothelial dysfunction by habitual aerobic exercise in healthy humans: Possible role of nuclear factor κB. Clinical Science, 127, 645–654. 24947434 10.1042/CS20140030 PMC4408779

[eph70288-bib-0074] Yang, A. C. , Stevens, M. Y. , Chen, M. B. , Lee, D. P. , Stähli, D. , Gate, D. , Contrepois, K. , Chen, W. , Iram, T. , Zhang, L. , Vest, R. T. , Chaney, A. , Lehallier, B. , Olsson, N. , du Bois, H. , Hsieh, R. , Cropper, H. C. , Berdnik, D. , Li, L. , … Wyss‐Coray, T. (2020). Physiological blood–brain transport is impaired with age by a shift in transcytosis. Nature, 583, 425–430. 32612231 10.1038/s41586-020-2453-z PMC8331074

[eph70288-bib-0075] Yew, B. , Jang, J. Y. , Dutt, S. , Li, Y. , Sible, I. J. , Gaubert, A. , Ho, J. K. , Blanken, A. E. , Marshall, A. , Shao, X. , Wang, D. J. J. , & Nation, D. A. (2022). Cerebrovascular reactivity deficits in cognitively unimpaired older adults: Vasodilatory versus vasoconstrictive responses. Neurobiology of Aging, 113, 55–62. 35325813 10.1016/j.neurobiolaging.2022.02.006 PMC10958374

[eph70288-bib-0076] Zhang, F. , Eckman, C. , Younkin, S. , Hsiao, K. K. , & Iadecola, C. (1997). Increased susceptibility to ischemic brain damage in transgenic mice overexpressing the amyloid precursor protein. Journal of Neuroscience, 17, 7655–7661. 9315887 10.1523/JNEUROSCI.17-20-07655.1997 PMC6793926

[eph70288-bib-0077] Zlokovic, B. V , Gottesman, R. F. , Bernstein, K. E. , Seshadri, S. , McKee, A. , Snyder, H. , Greenberg, S. M. , Yaffe, K. , Schaffer, C. B. , Yuan, C. , Hughes, T. M. , Daemen, M. J. , Williamson, J. D. , González, H. M. , Schneider, J. , Wellington, C. L. , Katusic, Z. S. , Stoeckel, L. , Koenig, J. I. , … Chen, J. (2020). Vascular contributions to cognitive impairment and dementia (VCID): A report from the 2018 National Heart, Lung, and Blood Institute and National Institute of Neurological Disorders and Stroke Workshop. Alzheimer's & Dementia: The Journal of the Alzheimer's Association, 16, 1714–1733. 10.1002/alz.12157 33030307

